# Intravenous Leiomyomatosis—A Single Centre Experience and Literature Review

**DOI:** 10.3390/reports9040333

**Published:** 2026-10-01

**Authors:** Olivera Dzatic Smiljkovic, Vesna Mandic Markovic, Ksenija Lalic, Lazar Nejkovic, Aleksandar Ristic

**Affiliations:** 1 Faculty of Medicine, University of Belgrade, Dr Subotica 8, 11000 Belgrade, Serbia; lola.dzatic.smiljkovic@gmail.com (O.D.S.); lazar.nejkovic@gmail.com (L.N.); 2 University Clinic for Gynecology and Obstetrics “Narodni front”, Kraljice Natalije 62, 11000 Belgrade, Serbia; ksenijal999@yahoo.com (K.L.); ristic.aleksandar@gakfront.org (A.R.)

**Keywords:** intravenous leiomyomatosis, uterine leiomyoma, intravenous extension, residual disease, recurrence, bilateral oophorectomy, multidisciplinary approach

## Abstract

**Background/Objectives:** Intravenous leiomyomatosis (IVL) is a rare, histologically benign proliferation of uterine smooth-muscle cells that extend intravascularly. The clinical significance of IVL lies in the potential for mechanical obstruction of the major veins and heart, as well as possible thromboembolic complications. **Methods:** A retrospective case series included four patients with confirmed IVL, treated at the Clinic for Gynaecology and Obstetrics “Narodni front” between 1 January 2020 and 31 December 2025. The following were recorded: medical history, imaging diagnostics (ultrasound, MRI), operative findings, histopathology, and follow-up data. The study was approved by the Ethics Committee, and written informed consent for participation in the study was obtained from all the patients. **Results:** The series included four patients aged 40 to 73 with histologically confirmed IVL who were treated surgically. In one patient, an intraoperative complication occurred in the form of sigmoid colon lesion and consequential resection, requiring surgical reconstruction, while the remaining procedures were completed without major complications. Histopathological findings in all cases indicated benign smooth-muscle differentiation with the presence of intravascular nodules of smooth-muscle cells (IVL). Residual disease and recurrence were documented in one patient. **Conclusions:** Our cases reflect a diverse spectrum of clinical presentations of IVL indicating the heterogeneity of the disease and the importance of a multimodal, multidisciplinary approach to the optimal management of this rare entity. The absence of further disease progression in our patients following bilateral oophorectomy, i.e., iatrogenic menopause, suggests the possible hormonal dependence of IVL.

## 1. Introduction

Intravenous leiomyomatosis (IVL) was first described 130 years ago by Birch-Hirschfeld [1]. It represents a histologically benign but potentially life-threatening proliferation of uterine smooth-muscle cells that typically extends from the venous vessels of the uterus, through the uterine veins and then through the lumen of the iliac veins, propagating upstream to various locations, such as the inferior vena cava (IVC), adrenal and renal veins, and the right side of the heart. Another route of extension is through the ovarian vein, directly into the IVC on the right side or into the left renal vein [2,3,4]. Intravascular extension of IVL may cause intracardiac obstruction or pulmonary tumour embolism, sometimes requiring urgent surgery [3].

IVL is often regarded as a variant of uterine leiomyoma [5]. While there is a strong consensus that oestrogen (ER) and progesterone (PR) promote the growth of uterine leiomyomas, i.e., that these tumours progress during the reproductive period and regress after menopause, intravenous leiomyomatosis is probably a hormone-sensitive tumour [5,6,7,8]. The leading hypotheses for histogenesis of IVL include extension of a pre-existing uterine myoma through the venous wall into the lumen and, alternatively, development from the smooth-muscle layer of the venous vessel wall itself [9].

A significant proportion of patients with IVL have a history of previous uterine surgery. The clinical presentation of the IVL is heterogeneous, ranging from asymptomatic incidental findings to symptomatic bleeding and pelvic pain, and to lower-extremity edoema, dyspnea, and syncope in cases of extension into the IVC or right atrium [10,11].

Accurate mapping of tumour continuity with cross-sectional imaging (contrast-enhanced CT and particularly MRI with angiographic reconstruction) and cardiac evaluation (TTE/TEE when intracardiac extension is suspected) is essential for surgical planning [12]. Surgical approaches vary and must be individualised. Clinical experience and case series suggest that the recurrence rate decreases when complete surgical excision (R0) is achieved and oophorectomy (iatrogenic menopause) is performed [5,8,13].

A definitive diagnosis of IVL requires demonstration of the tumour within the lumen of a vein and, when it is available, in correlation with radiological evidence of intravascular extension [5]. Given the possibility of disease recurrence, follow-up protocols include long-term surveillance and defined criteria for radiological monitoring [5,13,14,15,16].

In this study, we retrospectively analysed four cases of histologically confirmed intravenous leiomyomatosis and systematically presented their medical histories, diagnostic findings, surgical approaches, and treatment outcomes, aiming to contribute to the monitoring and understanding of this rare disease.

## 2. Materials and Methods

This retrospective descriptive case series included four patients with histologically confirmed intravascular leiomyomatosis (IVL) who were treated at the Clinic for Gynecology and Obstetrics “Narodni front” (CGO NF) between 1 January 2020 and 31 December 2025. Patients were included sequentially by the date of diagnosis. These four patients represent all histopathologically confirmed IVL cases at our centre during the study period. The Ethics Committee of CGO NF granted institutional approval for the study (Approval No. 22008-2026-008015 from 27 April 2026) in accordance with internationally accepted ethical standards (The Helsinki Declaration of 1964, as revised in 1975, 1983 and 1989). All participants provided informed written consent for the use and publication of anonymized data.

The inclusion criteria were histopathological confirmation of IVL, available preoperative documentation, and clinical data documented in the medical records. Data were collected retrospectively by reviewing hospital records in the Heliant and IZIS systems and through medical history obtained during interviews with the patients. Patients had variable follow-up durations (from 2 to 67 months). Follow-up was measured from the date of the first to the date of the last clinical contact.

For each patient, demographic data, relevant medical history, clinical presentation, detailed imaging findings (US, MRI), types of surgical procedures, intraoperative findings, histopathological reports (macroscopic findings, microscopic findings, immunohistochemistry (IHC)), and postoperative outcomes and follow-up data were recorded. Demographic and clinical characteristics, treatment details, and follow-up outcomes for all patients are summarised in tables. Additional relevant findings are provided in the text.

Representative imaging and histopathological findings are illustrated as figures within the manuscript. Available MRI scans were retrospectively evaluated by the clinical radiologist from our clinic.

Residual disease was defined as macroscopic or histologically confirmed tumour remaining at the surgical site immediately after the operation or identified on early postoperative imaging. Radiological progression was defined as an increase in size or extent of known residual intravascular tumour compared with prior imaging, measured by objective criteria (e.g., longest diameter or volumetric increase). Recurrence was defined as development of new intravascular tumour lesions at sites previously free of disease after documented complete resection (i.e., no residual disease on immediate postoperative imaging). Reintervention was defined as any additional surgical or endovascular procedure performed with the intent to treat persistent or progressive intravascular disease.

The primary outcomes were postoperative complications, the need for reoperation, and disease status at the last follow-up. Due to the small number of cases, the data analysis was descriptive. Quantitative data are presented as median and range (min–max).

## 3. Results

We report four patients (age range 40–73 years, median 46.5 years) who presented with histopathological evidence of intravascular leiomyomatosis (IVL). Clinical presentations included heavy menstrual bleeding with resulting anaemia, pelvic pressure or urinary symptoms. All patients underwent surgical treatment. Key clinicopathologic features, management decisions and outcomes are summarized in the text and tables below, and also in Supplementary Table S1 [Table 1, Table 2 and Table 3].

### 3.1. Case 1

**Figure 1:**
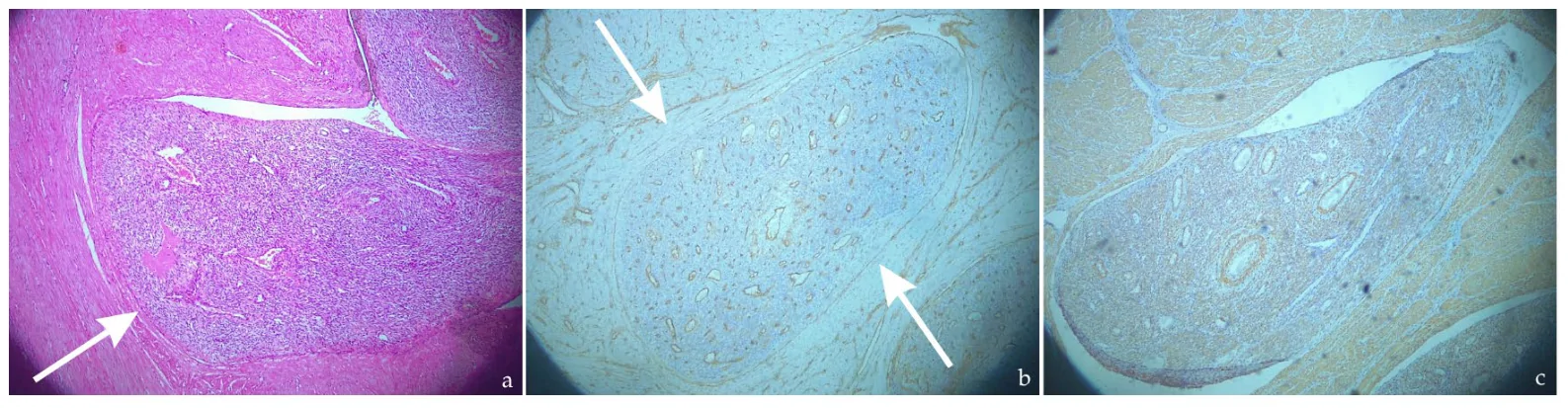
(**a**) Intravascular proliferation of smooth-muscle cells (H&E, original magnification ×5); (**b**) smooth muscle bundles filling a vascular space, forming a well-circumscribed intravascular nodule (H&E, original magnification ×5); (**c**) immunohistochemical staining shows H-caldesmon-positive smooth-muscle cells with diffuse, strong H-caldesmon expression and tumour nodules present within the lumina of blood vessels (original magnification ×5).

A 40-year-old woman with a long history of large and multiple uterine fibroids presented with progressive heavy menstrual bleeding, anaemia and pollakiuria. Her prior interventions included laparoscopic myomectomy (2015) and, after that, two curettage procedures for heavy bleeding. Histopathological findings from those procedures were unremarkable. In late 2020 she underwent hysterectomy with bilateral salpingectomy and conservation of ovaries. The procedure was complicated by extensive adhesions in the pelvic cavity, with the sigmoid colon densely adherent to pelvic peritoneum from previous surgery. During adhesiolysis, a lesion of the sigmoid colon occurred. For that reason, a surgeon was consulted intraoperatively and a segmental sigmoid resection with immediate end-to-end anastomosis was performed. Histopathologic finding established the diagnosis of IVL [Figure 1].

Immunohistochemical staining was performed and the results were consistent with IVL [Figure 1].

**Figure 2:**
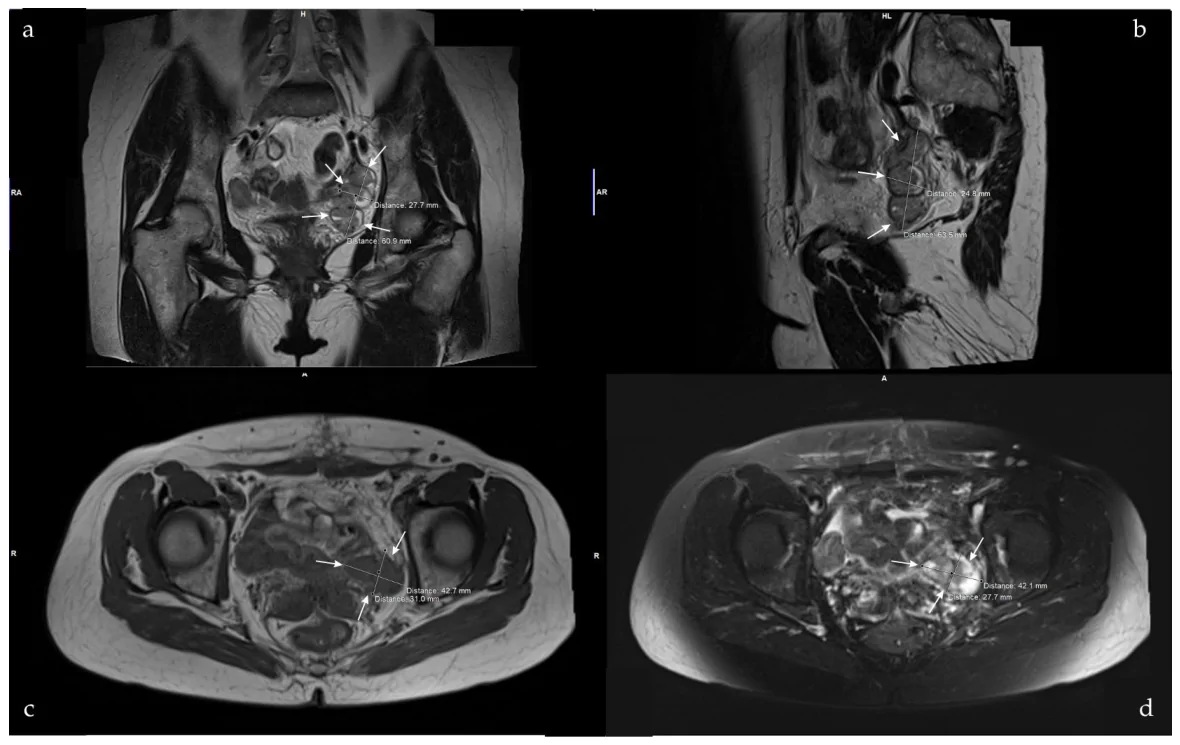
First MRI after hysterectomy with bilateral salpingectomy and conservation of ovaries (October 2021) with multiple mostly solid nodules on the left side (60.9 × 27.7 mm); All arrows indicate the same lesion. (**a**) T2w TSE coronal plane (60.9 × 27.7 mm); (**b**) T2w TSE sagital plane; (**c**) T1w TSE axial; and (**d**) T2w Fs axial.

One month postoperatively, pelvic MRI demonstrated multiple predominantly solid nodules in the left adnexal region. The lesions were described as probably retroperitoneal, well-circumscribed and encapsulated, with strong enhancement after IV contrast—findings suspicious for residual tumours [Figure 2].

Smaller solid lesions were also identified in the right adnexal region, which could have corresponded to an altered ovary or an additional tumour nodule.

Following histopathological confirmation of intravenous leiomyomatosis, the patient was reviewed by the oncology board of our clinic. Given MRI evidence of probable residual disease and the tumour’s nature, BO was recommended.

**Figure 3:**
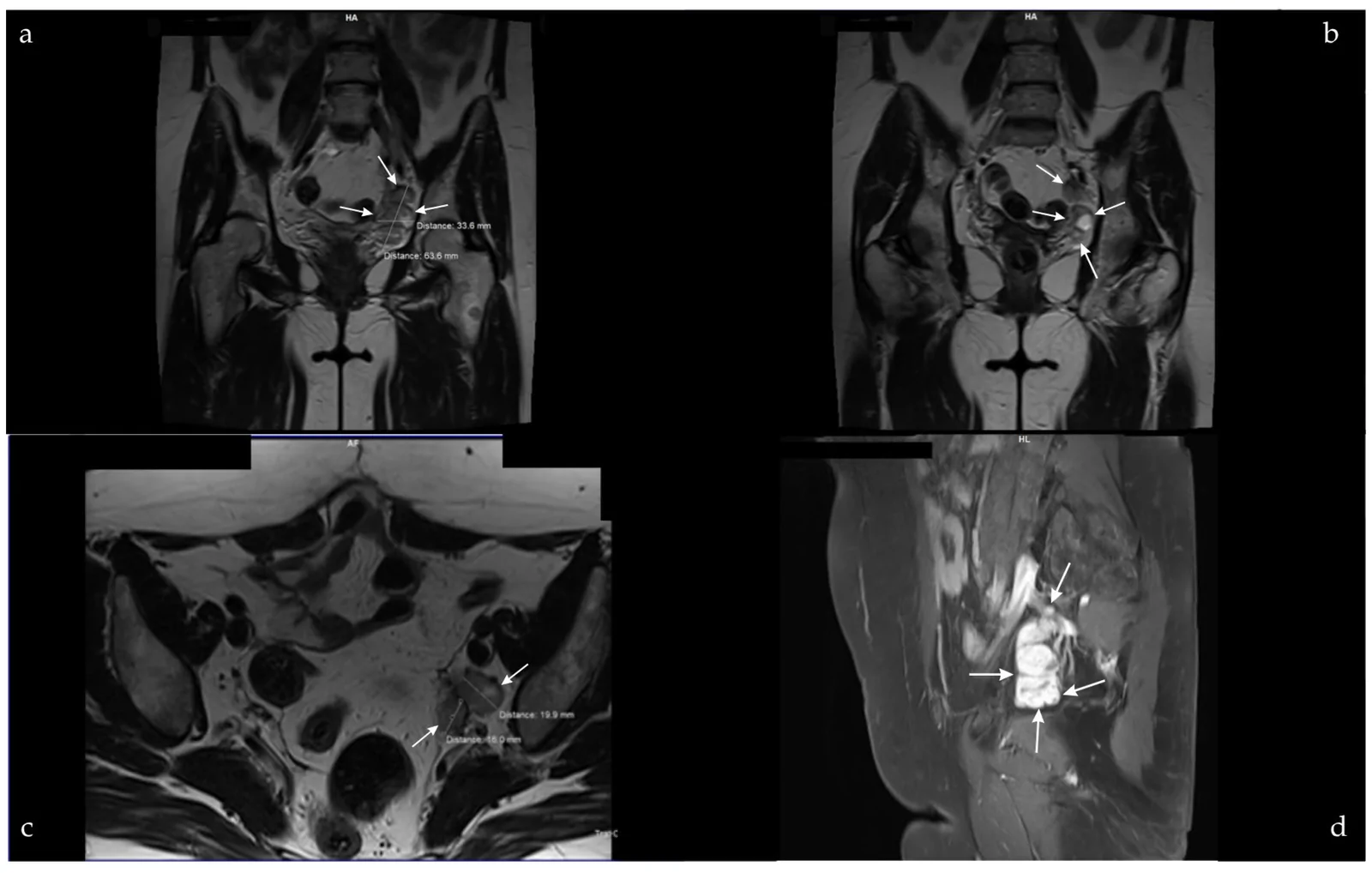
MRI October 2021; multiple solid nodules on the left side; All arrows point to the described lesion; (**a**) T2w TSE coronal plane (63.6 × 33.6 mm); (**b**) T2w TSE coronal plane; (**c**) T1w TSE axial plane; and (**d**) T1w TSE sagittal plane.

Several months later (May 2021) BO with adhesiolysis and excision of a 2 × 1 cm lesion from the vaginal cuff (histopathologically leiomyoma—recurrence) was performed. Supplementary IHC showed moderate-to-strong nuclear ER and PR expression in >90% of tumour cells. Subsequent imaging documented multiple intra- and retroperitoneal solid nodules in the left adnexal bed and parailiac region, which were slightly larger compared to the previous MRI. Abnormalities on the right side were not described. Reports recommended surgical correlation in consultation with a gynaecologist and vascular surgeon [Figure 3].

**Figure 4:**
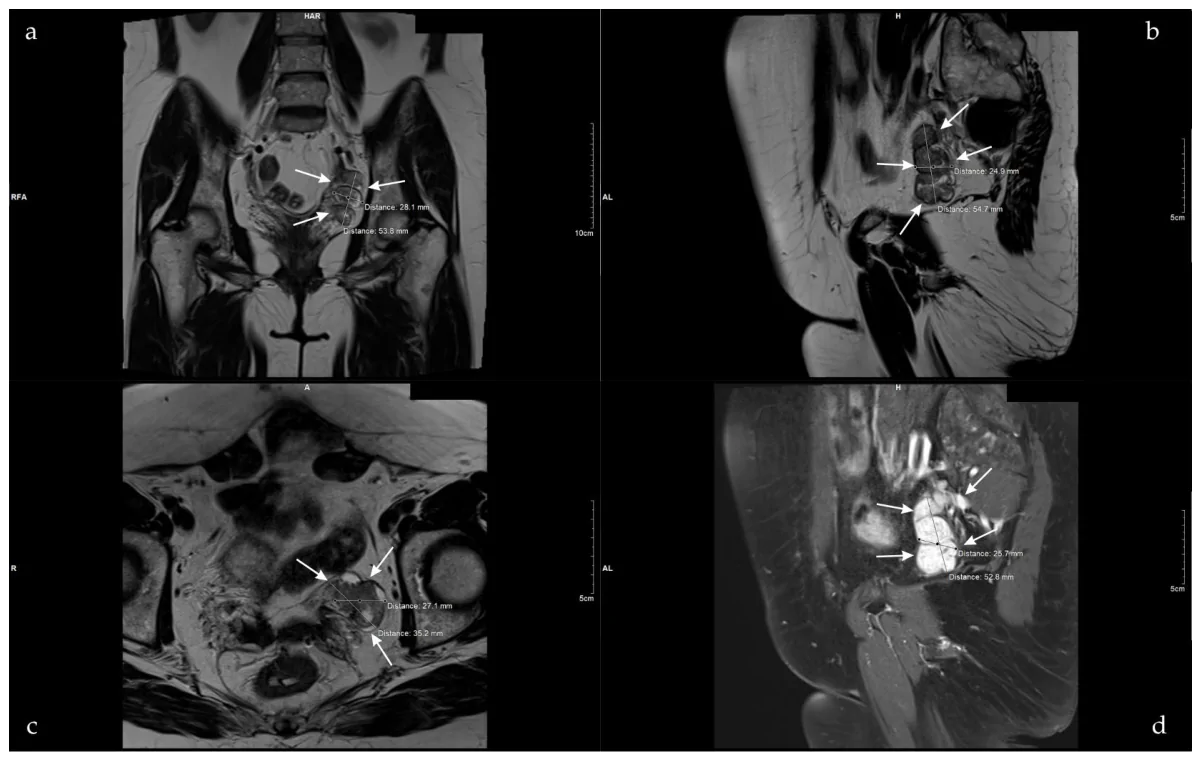
MRI March 2022; multiple solid nodules on the left side; All arrows point to the described lesion; (**a**) T2w TSE coronal plane (53.8 × 27.1 mm); (**b**) T2w TSE sagital plane; (**c**) T2w TSE axial plane; and (**d**) T1w TSE sagittal plane.

The patient declined further surgery and was managed conservatively. The finding on the most recent MRI compared with the previous one was stable and even showed slight regression [Figure 4].

**Figure 5:**
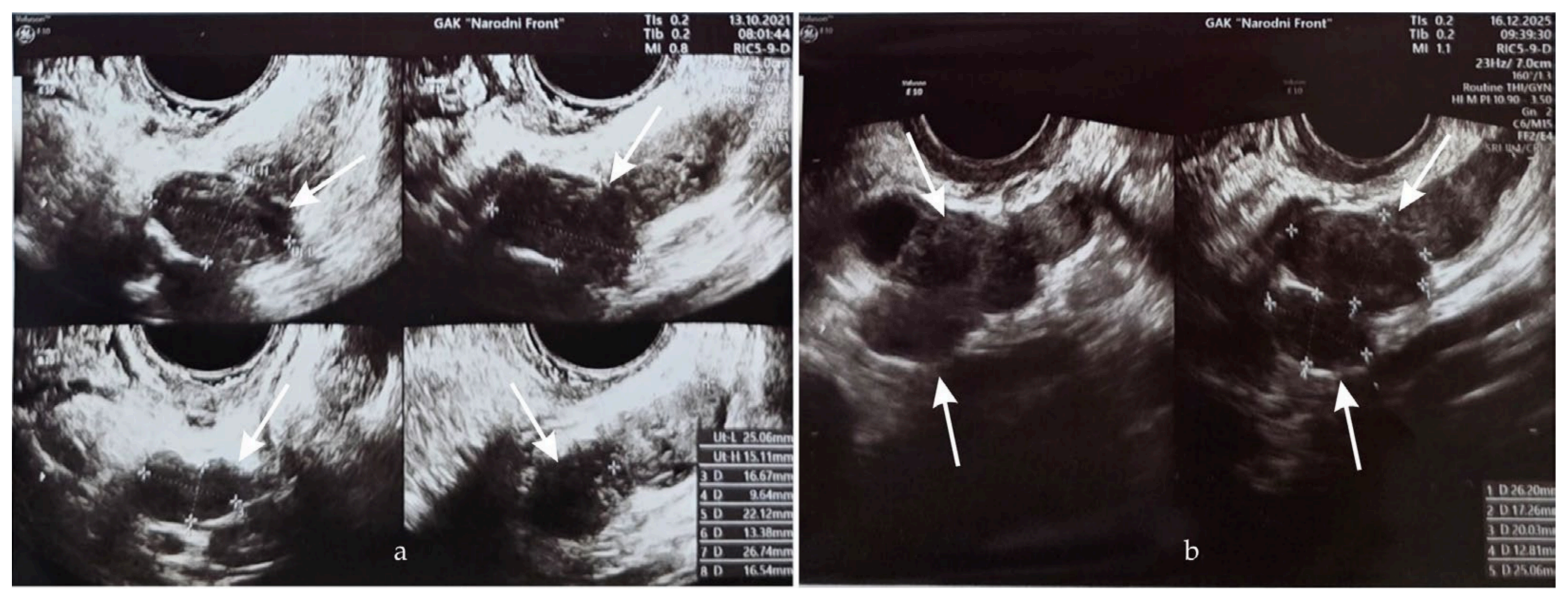
US findings showed no signs of progression; All arrows point to the described lesion; (**a**) few months after BO; (**b**) last follow-up (2025).

To date, US findings after the BO showed no sign of progression [Figure 5].

Additional postoperative imaging, including contrast-enhanced CT and CT angiography, was requested on multiple occasions during the follow-up. The patient did not undergo these examinations.

The MRI appearance, the patient’s asymptomatic status, and stable US examinations suggested no disease progression; however, continued imaging surveillance is recommended.

### 3.2. Cases 2–4

Three patients underwent hysterectomy with bilateral adnexectomy (Cases 2 and 3) or hysterectomy with ipsilateral adnexectomy and salpingectomy after prior unilateral oophorectomy (Case 4). Our second patient was a 73-year-old postmenopausal patient who underwent vaginal hysterectomy with anterior/posterior vaginal repair for pelvic organ prolapse. She stated that she was unaware of the presence of uterine fibroids priorto her hospitalisation at the clinic. Following surgery, histopathology revealed multiple myometrial nodules with intravascular spindle-cell aggregates consistent with dissecting leiomyoma with IVL. Case 3, a 47-year-old patient undergoing prophylactic surgery during breast-cancer treatment, had a history of long-standing intramural leiomyoma. She reported a history of heavy menstrual bleeding and, prior to surgery, pollakiuria. Histopathology showed intravascular spindle-cell nodules. Case 4, a 46-year-old patient with heavy menstrual bleeding, anaemia and sensations such as pressure and pain in the lower abdomen, had a large (15 × 20 cm) retroperitoneal mass displacing the uterus, excised en bloc with the uterus and right adnexa. A pathologist who was consulted intraoperatively reported that the lesion was consistent with a leiomyoma based on its morphological characteristics. Histopathological finding confirmed IVL. Postoperative US imaging was unremarkable for all three patients; further follow-up is planned (median planned interval: 6 months).

## 4. Discussion

In our series of four patients (age range 40–73 years; median 46.5 years), the demographic distribution and clinical behaviour were typical for IVL. Three patients developed iatrogenic menopause following BO, while one patient had already been postmenopausal for 20 years at the time of IVL diagnosis. Three patients had a history (>10 years) of uterine fibroids, consistent with reported coexistence of IVL and leiomyomas [17,18]. The clinical presentation, including abnormal uterine bleeding and resulting anaemia, as well as a sensation of pressure and pollakiuria (due to local compression of adjacent structures by the fibroids), is typical of patients with large or multiple fibroids, as well as of those with IVL [16]. Three of our patients presented with this clinical pattern.

In Cases 2 and 3 the clinical presentation was considerably milder than in the other two patients, and there was no evidence of extension of IVL beyond the uterus. Despite the absence of more severe clinical findings, the diagnosis of IVL is based on histopathology. Radiologic surveillance remains important for monitoring possible progression into larger venous channels and distant branches [5,19].

The literature evidence for hormonal dependence of intravenous leiomyomatosis (IVL) to date remains limited. Liang et al. reported ER and/or PR positivity in all 15 intracardiac IVL cases, and oestrogen-deprivation therapy reduced tumour burden and enabled complete resection in some initially unresectable patients. Ovarian preservation after incomplete resection was linked to progression or recurrence in up to 75% of premenopausal cases [8]. Hormonal therapies (GnRH agonists or aromatase inhibitors) can be beneficial, but effectiveness depends on tumour hormonal status and patient tolerance. Decisions on oophorectomy versus conservative management with adjuvant therapy should be individualised within a multidisciplinary team with clear patient counselling [11,13,20,21].

In Case 1 the immunohistochemical findings (SMA+/desmin+, low Ki-67) indicate benign smooth-muscle differentiation, yet do not differentiate IVL from a conventional leiomyoma [22,23]. Nuclear expression of ER and PR receptors, which was observed in >90% of tumour cells, and the absence of disease progression after bilateral oophorectomy in this case is consistent with the hypothesis of hormonal dependence of IV [15,24]. Oophorectomy was performed as a second-stage procedure after radiologically confirmed residual disease; the ovaries were preserved initially to maintain fertility. Optimal IVL management includes detailed pelvic radiological mapping, multidisciplinary tumour-board planning, and R0 resection (not always technically feasible), which are associated with lower recurrence rates [16,20]. Residual disease, multifocality, and firm vascular adherence of the tumour increase recurrence risk [5,8,17,21,25]. Induction of menopause by oophorectomy may reduce hormone-dependent progression of residual tumour, but it does not definitely prevent recurrence especially in the presence of residual or distant disease [13,17,25]. Therefore, adjuvant hormonal therapy and intensified radiological surveillance should be considered [14,15,16,26]. In our series, no progression was observed during follow-up in the remaining three cases. The fact that all our patients were postmenopausal is consistent with, but does not constitute definitive proof of, hormonal sensitivity.

The surgical outcomes in our series illustrate the diverse clinical presentations of IVL. In one case, the procedure was complicated by sigmoid colon injury, while another case required additional excision of a retroperitoneal mass. These findings confirm that IVL may require a multidisciplinary approach (involving gynaecology, vascular surgery, abdominal surgery, and interventional radiology) and that preoperative planning is essential for reducing intraoperative complications and optimising outcomes [9].

Nevertheless, all these findings must be interpreted cautiously. The small sample size and retrospective design limit the statistical power and generalisability of the results. The relatively short duration of follow-up (especially in Cases 3 and 4) may underestimate rates of late recurrence, which is particularly important for tumours with potential hormone sensitivity and indolent growth. Although >90% ER/PR positivity in a single case is notable, the absence of systematic molecular profiling (e.g., CNV analysis, MED12 and other genetic/epigenetic assessments) prevents correlation of immunohistochemical findings with clinical course. Additionally, heterogeneity and variable availability of radiological imaging across patients limit comparability of findings. Stronger conclusions on this topic would require larger, ideally prospective studies with longer follow-up and integrated molecular analyses. Future research should consider prospective studies in selected younger women with IVL to evaluate the potential role of conservative treatment strategies, including adjuvant hormonal therapy, within a multidisciplinary approach.

## 5. Conclusions

Our cases reflect a diverse spectrum of IVL clinical presentations. These patterns are consistent with published case series demonstrating the heterogeneity of the disease and the importance of a multimodal, multidisciplinary approach to the optimal management of this rare entity. The fact that there was no disease progression following BO in our group of patients is compatible with the hypothesis that IVL may be hormonally influenced. However, study limitations preclude firm causal conclusions.

## Figures and Tables

**Table 1:** Clinical and demographic characteristics of patients.

Case ID	Age	Age at Diagnosis of Uterine Myomas	G/P *	Clinical Presentation	Previous Procedures	Preoperative Imaging	Follow-Up Duration (Months)
1	40	27	0/0	Menometrorrhagia, anaemia, pollakiuria (without UTI)	Laparoscopic myomectomy	Ultrasonography	67
2	73	73	3/2	Stress urinary incontinence	None	Ultrasonography	21
3	47	26	2/2	Menorrhagia, pollakiuria	Right-sided radical mastectomy	Ultrasonography	2
4	46	35	2/2	Menorrhagia, anaemia, fatigue, pressure in the lower abdomen	Left oophorectomy due to endometriosis	Ultrasonography, MRI	3

* G/P—Gravida/Para.

**Table 2:** Surgery, complications and resection status.

Case ID	Initial Surgical Procedure	Intraoperative Findings	Intraoperative Complications	Resection Status Evaluated by US/MRI
1	Hysterectomy with bilateral salpingectomy	The pelvis was occupied by a 20 cm tumour mass, with several smaller myomas at the uterine fundus and in the isthmic region; the bowel was adherent to the peritoneum of the pelvic organs	Sigmoid colon injury, resection, and end-to-end anastomosis	Incomplete (MRI)
2	Total vaginal hysterectomy with bilateral adnexectomy; anterior and posterior colporrhaphy	Unremarkable	None	Complete (US)
3	Total laparoscopic hysterectomy with bilateral adnexectomy	Uterus myomatosus	None	Complete (US)
4	Hysterectomy with right adnexectomy and left salpingectomy with excision of the tumour mass	A retroperitoneal tumour mass displacing the uterus was identified. The right adnexa were adherent to the uterus.	None	Complete (US)

**Table 3:** Histopathology, immunohistochemistry and follow-up status.

Case ID	Histopathology	Immunohistochemistry	Atypia or Increased Mitotic Activity	Status at Last Follow-Up
1	Macroscopic finding—the uterus was markedly enlarged and with a large tumour (140 × 110 × 100 mm) on the posterior wall, extending into the uterine cavity; multiple additional intramural and subserosal fibrous nodules were present. Microscopic finding—leiomyomatous nodules within tthe myometrium and outside the myometrium nodules of spindle cells within dilated venous channels—histomorphology and IHC identical to the main tumour.	Desmin, alpha-SMA, H-caldesmon (strong, diffuse expression in the tumour nodules within the blood vessel lumens); Ki-67 3%; moderate to strong nuclear ER and PR expression in >90% of tumour cells	No	No evidence of disease progression
2	Macroscopic and microscopic examination revealed multiple myometrial tumour nodules with intravascular smooth-muscle nodules.	CD34 positive in the endothelial cells of the blood vessels	No	Ultrasound examinations performed after one month and five months were unremarkable
3	Macroscopic examination revealed greyish-white area (3 cm) within the myometrium. Nodules of smooth-muscle tissue were observed within individual blood vessels in the surrounding myometrium.	Not reported	No	Ultrasound examination performed one month after surgery was unremarkable
4	Macroscopic examination revealed numerous greyish-white, firm nodules within the myometrium, as well as a single subserosal nodule of similar appearance, measuring approximately 6 cm. The nodules also appeared to be present within the lumen of blood vessels. A separate tumour specimen was submitted, measuring 19 × 10 × 6 cm, greyish-white in colour, firm in consistency, and lobulated in appearance. Microscopically, nodules of tumour tissue composed of bundles of uniform spindle cells were observed in numerous blood vessels.	Not reported	No	Ultrasound examination performed one month after surgery was unremarkable

## Data Availability

Data presented in this study are available from the corresponding author upon request.

## Supplementary Materials

The following supporting information can be downloaded at https://www.mdpi.com/article/10.3390/reports9040333/s1, Table S1: Characteristics of the patients with IVL.

## Abbreviations

The following abbreviations are used in this manuscript:

IVL	Intravenous leiomyomatosis
CGO NF	Clinic for Gynaecology and Obstetrics “Narodni front”
US	Ultrasound
ER	Oestrogen
PR	Progesterone
BO	Bilateral oophorectomy
IVC	Inferior vena cava
CT	Computed Tomography
MRI	Magnetic resonance imaging
TTE	Transthoracic echocardiography
TEE	Transoesophageal echocardiography
IHC	Immunohistochemistry
UTI	Urinary tract infection
