# Supplementary material for: Intravenous Leiomyomatosis—A Single Centre Experience and Literature Review
Source: Reports (MDPI). 2026 Oct 1;9(4):333. doi: 10.3390/reports9040333 (PMC13692487; doi:10.3390/reports9040333)
Supplement: Supplementary file 1 [file reports-09-00333-s001.zip › reports-4581029-supplementary.pdf]

Supplementary Table S1: Characteristics of the patients with IVL

Case ID	1	2	3	4
G/P	0/0	3/2	2/2	2/2
Menopause	Iatrogenic	Over 20 years ago	Iatrogenic	Iatrogenic
Previous gynaecological procedures	Laparoscopic myomectomy Curretage	/	Right-sided radical mastectomy (breast cancer)	Left oophorectomy due to endometriosis
Comorbidities	Insulin resistance, anaemia	Hypertension, rheumatoid arthritis	Hypertension, cardiac arrhythmia	Hypertension, anaemia
Family history relevant to uterine myomas	Four relatives had uterine myomas with heavy menstrual bleeding	/	Mother had heavy menstrual bleeding	/
Age at diagnosis of uterine myomas	27	73	26	35
Clinical presentation	Menometrorrhagia, anaemia, pollakiuria (without UTI)	Stress urinary incontinence	Menorrhagia	Menorrhagia, anaemia, fatigue, pressure in the lower abdomen
Age at diagnosis of IVL	39	73	47	46
Ultrasound findings	Multiple uterine myomas	Uterine myoma displacing the uterine cavity	Myomatous uterus	Anteuterine myomatous lesion
MRI of the abdomen and pelvis	MRI revealed residual disease	Not performed	Not performed	Preoperatively – Well-defined subserosal bigger myoma in the anterior uterine wall with an additional left fundal smaller myoma**.**
Farthest extent of the tumour	Pelvis and abdomen	Myometrium	Myometrium	Pelvis
Maximum lesion size	20 cm	3.2 cm	3 cm	19 cm
First surgery at our Clinic	Hysterectomy with bilateral salpingectomy; Adhesiolysis; Sigmoid colon resection, and end-to-end anastomosis	Total vaginal hysterectomy with bilateral adnexectomy; Anterior and posterior colporrhaphy	Total laparoscopic hysterectomy with bilateral adnexectomy	Hysterectomy with right adnexectomy and left salpingectomy with excision of the tumour mass
Intraoperative findings	The pelvis was occupied by a 20 cm tumour mass, with several smaller myomas at the uterine fundus and in the isthmic region; the bowel was adherent to the peritoneum of the pelvic organs	Unremarkable	Unremarkable,	Upon entering the abdominal cavity, an anterior uterine tumour mass was identified, displacing the uterus; the right adnexa were adherent to the uterus
Histopathological findings	A benign tumour was described microscopically – a dissecting intramural and submucosal leiomyoma. In the myometrium nodules of spindle cells identical to those of the tumour were present outside the boundaries of the described tumour, and within dilated venous blood vessels	The largest grayish-white nodule in the myometrium measured approximately 3 cm in diameter. Nodules of smooth muscle tissue were observed in individual blood	A partially lobulated grayish-white area in the myometrium, measuring 3 cm in diameter. In the surrounding tissue, nodules of smooth muscle tissue were observed within individual blood vessels	Sections taken from the tumour nodules in the myometrium, as well as from numerous blood vessels, revealed nodules of tumour tissue composed of bundles of uniform spindle cells and blood vessels with thickened walls

		vessels of the surrounding myometrium		
Immunohistochemistry	Desmin, alpha-SMA, H-caldesmon (strong, diffuse expression in the tumour nodules within the blood vessel lumens); Ki-67 approximately 3%; moderate to strong nuclear ER and PR expression in >90% of tumour cells	CD34 positive in the endothelial cells of the blood vessels	Not reported	Not reported
Second surgery at our Clinic	Bilateral oophorectomy; Adhesiolysis	Not performed	Not performed	Not performed
Age at the time of surgery	39	N/A	N/A	N/A
Intraoperative findings	The omentum was adherent to the peritoneum of the anterior abdominal wall and to the sigmoid colon, which was adherent to both ovaries. A tumor mass measuring approximately 2 cm in diameter was present at the vaginal cuff	Not performed	Not performed	Not performed
Residual disease and/or Recurrence	Yes	No	No	No
Follow-up	The patient declined further surgical treatment; the findings have remained stable over the past four years. Annual follow-up is planned	Follow-up ultrasound examinations after one month and four months - unremarkable; the next follow-up examination is scheduled in one year	Follow-up ultrasound examination one month after surgery was unremarkable; further follow-up examinations are planned every six months	Follow-up ultrasound examination one month after surgery was unremarkable; the next follow-up examination is scheduled for six months after surgery
